# Community participation to design rural primary healthcare services

**DOI:** 10.1186/1472-6963-14-130

**Published:** 2014-03-21

**Authors:** Jane Farmer, Amy Nimegeer

**Affiliations:** 1 La Trobe Rural Health School, La Trobe University, Bendigo, Victoria 3552, Australia; 2 School of Nursing, Midwifery and Health, University of Stirling, Stirling FK9 4LA, Scotland

**Keywords:** Community participation, Primary health care, Rural health, Healthcare reform, Community engagement, Co-production, Population health planning

## Abstract

**Background:**

This paper explores how community participation can be used in designing rural primary healthcare services by describing a study of Scottish communities. Community participation is extolled in healthcare policy as useful in planning services and is understood as particularly relevant in rural settings, partly due to high social capital. Literature describes many community participation methods, but lacks discussion of outcomes relevant to health system reconfiguration. There is a spectrum of ideas in the literature on how to design services, from top-down standard models to contextual plans arising from population health planning that incorporates community participation. This paper addresses an evidence gap about the outcomes of using community participation in (re)designing rural community health services.

**Methods:**

Community-based participatory action research was applied in four Scottish case study communities in 2008–10. Data were collected from four workshops held in each community (total 16) and attended by community members. Workshops were intended to produce hypothetical designs for future service provision. Themes, rankings and selections from workshops are presented.

**Results:**

Community members identified consistent health priorities, including local practitioners, emergency triage, anticipatory care, wellbeing improvement and health volunteering. Communities designed different service models to address health priorities. One community did not design a service model and another replicated the current model despite initial enthusiasm for innovation.

**Conclusions:**

Communities differ in their receptiveness to engaging in innovative service design, but some will create new models that fit in a given budget. Design diversity indicates that context influences local healthcare planning, suggesting community participation impacts on design outcomes, but standard service models maybe useful as part of the evidence in community participation discussions.

## Introduction

This paper explores the outcomes from inviting community members to participate in designing primary healthcare services for remote rural places. In our study, healthcare models were designed that address communities’ priorities and are affordable within existing budgets. The community participation process we used involves local people in service decision-making as is desired within contemporary healthcare policy.

Internationally, remote and rural places are changing due to conditions of global capitalism [1]. One result is that younger, working age people become concentrated in metropolitan and commuter areas, leaving concentrations of older people in smaller, more peripheral, remote rural settlements [2]. With regard to services, larger regional centres tend to have a range of primary healthcare and a general hospital, while smaller towns might have a hub of more limited primary healthcare services and a community hospital. Suitable service arrangements for small remote settlements are hard to define [3] and remote places are vulnerable to small changes in population and healthcare providers.

Contemporary advice on how to design primary healthcare services spans a range from providing rational, algorithmic models that suggest workforce based on accessibility to services and amenities [4] or need, combined with evidence of effectiveness [5] through to suggesting population health planning founded on discussing local priorities in relation to need and social determinants [6]. While there is strong direction from governments internationally to use community participation in local service planning [7,8], there is little clarity about what to do, what outcomes to expect and how to incorporate evidence about health innovation [9].

This paper shows outcomes from deploying a community participation process to design healthcare for remote communities. It draws on findings from a study ‘Remote Service Futures’ (RSF), the primary purpose of which was to devise a feasible methodology for remote community members’ participation in health service reconfiguration [10]. In 2008–10, within the action research process to devise the methodology, residents of four remote Scottish Highland communities were invited to participate in planning to identify their local health priorities and design ways to address these. This paper outlines the community participation methodology derived and the healthcare designs produced.

## Background

The Scottish Government Urban–rural Classification defines the settlements included in this study as “very remote rural areas” [11]. For shorthand, we call them remote. The Scottish Government defines very remote rural areas as having fewer than 3,000 inhabitants and being over an hour from a settlement of 10,000 or more. There are different categorisations and understandings of the concepts of remote and rural depending on countries’ size, population and geography, but we suggest that, internationally, remote areas share features of sparse population, distance from and therefore inaccessibility to, specialised services [12] and choice of services, and identification as geographically peripheral in the national psyche.

Relative to other UK states, Scotland is well resourced with general practitioners (GPs) and nurses [13] and these are more evenly distributed throughout the country than in other nations [14]. Remote health services are provided by a mix of GPs (either employed as National Health Service (NHS) independent contractors or salaried), community staff employed by regional health authorities, Scottish Ambulance Service personnel, council and voluntary/non-profit organisation workers. NHS24 is a national first response phone triage service and there are after-hours service schemes to see a primary healthcare practitioner. About 6.4% of Scotland’s 5.3 million population live in remote areas [15]. Remote and rural areas are distinguished by higher suicide rates, incidence of alcohol related disease, numbers of accidents and palliative care workload [16]. Compared with its urban areas, remote Scotland does not have severe socio-economic disadvantage, but there are pockets of disadvantage [17].

Internationally, problems of remote and rural healthcare include centralisation, lack of chronic condition care, health worker shortage, failure to adequately address prevention and lack of infrastructure for co-ordinated, integrated care [18]. Decades of policy to incentivise rural workforce and introduce tele-health have had very small impacts in the face of systemic and societal disincentives. Those concerned with redesigning healthcare for remote areas have had to formulate creative ideas about service provision and planning. At the time the RSF study started, a policy document *Delivering for Remote and Rural Healthcare* (2008) [19] had recently been produced and its goal was to provide a standard framework for how Scottish rural health services would be delivered into the future. It was the culmination of several years of discussion, some of it based on evidence from the NHS *Remote and Rural Areas Resources Initiative* which had been established in 2000 to address the problems of recruiting and retaining rural health professionals.

There is a spectrum of approaches to deciding what health services a rural community should have. One way is to provide standard service designs for types of places with certain packages of objective characteristics. In Australia, a rural and remote primary healthcare typology has been established, with authors concluding that “ a critical minimum population base of about … 2,000-3,000 people for remote communities is necessary to support a comprehensive and sustainable range of … services” [3]. How to service communities of less than 2,000 people is not addressed. The Index of Rural Access applies a fine-grained approach to remote and rural healthcare planning [4], including service availability and proximity, population health needs and mobility and claims to be sensitive in describing accessibility deficits. Another algorithmic approach combines need and best practice evidence to design community workforce.

Using chronic disease as an exemplar, it identifies need for condition sub-populations, effective interventions and then calculates the competencies required for the local population [5].

An alternative to standardised models is to customise services to local context within a population health planning philosophy [20], incorporating community participation. Community participation has been described as “…social interactions to influence and localise outcomes” [21]. Variations are community engagement or involvement, which we consider are all about desire to include the views of local people in service planning. Keleher [6] describes population health planning as requiring stakeholder input, being predicated on a social determinants understanding and informed by data about care delivery, illness prevention, health promotion, resourcing and effective design and implementation. Community participation has been described as a ‘social process’ and ‘an ideal’ [22]. It has also been discussed as a state on a continuum between community readiness and community empowerment [23]. Experts [21,24] have commented on the need to distinguish between clients and citizens in participation, with clients’ participation based on self-interest as consumers, while citizen participation asks people to reach beyond their own concerns, to what is good for the community as a collective. The way we conceptualise community participation is as an intervention that could extend into a philosophy of working, with the key idea being that it endeavours to bring together the voices of those who have an interest in the community’s health – i.e. it’s a space in which local citizens, health and service practitioners and managers can discuss local health issues and how to address them.

Diverse motivations have been ascribed to why international governments promote community participation. The Scottish Government [25] describes need for:

“…a relationship where patients and the public are affirmed as partners rather than recipients of care… where we think of the people of Scotland not just as consumers – with only rights – but as owners – with both rights and responsibilities”.

Here, the Scottish Government is invoking both client/consumer (individual) and citizen/community (for the good of society), perspectives [21,24]. A review found over 100 methods for public engagement [26], including focus groups, participatory appraisal, Planning for Real, citizen’s juries and future visioning. With imprecision about what it is and many methods offered for doing it, it is unsurprising that health managers wrestle with understanding what to do about community participation. While there is general evidence about beneficial effects, few studies evaluate outcomes affecting health service decision-making [27].

Remote and rural settings have been suggested as prime sites for community participation due to a history of rural community development [28]. The OECD [2] has promoted community participation within the “new rural paradigm”, suggesting its centrality to harnessing rural communities’ internal resources. Rural places have been shown to have high social capital [29] and volunteering [30].

While community participation is an established policy concept, it has been suggested that little power or creative input into design has actually shifted to citizens [31]. Some in rural health think that the public would have difficulty making “realistic” decisions [32], presumably because understanding the health system is complex. Given the lack of studies showing whether or how community participation can affect service design, this paper offers new insights and starts to address a gap in knowledge.

## Methods

### Study design

**Figure 1:**
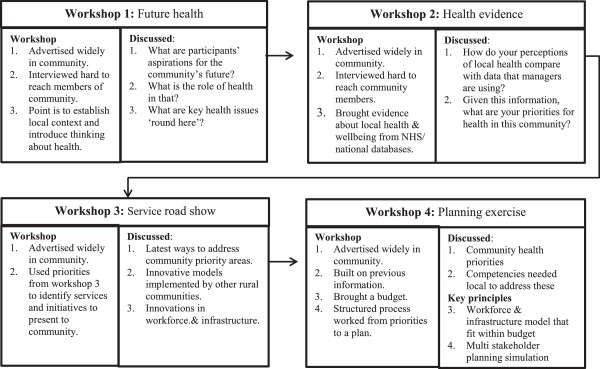
Community participation process.

The Remote Service Futures (RSF) study developed and applied a community participation process using community based participatory action research (CBPAR) [33]. Community members from four remote settings were invited to participate in four workshops. To include those who would not, or could not, attend workshops, face-to-face individual interviews, email and telephone conversations were also used. The process philosophy was to encourage community members to ask questions about local health and health services, to provide evidence in response, to inform priority-setting and service design, and to include health practitioners and managers as part of the discussion and sharing of evidence. Action research is used to address complex real-world problems by applying cycles of fact-finding, action and reflection [34]. CBPAR involves citizens as intelligent co-participants in tackling research problems, leading to “self-critical communities” [35], community capacity and co-learning between participants [33]. In RSF, a review of international literature informed design of a prototype community participation process. Action research applied, developed and refined the process (see Figure 1). Importantly, although RSF was intended to develop new primary healthcare models, local residents were informed this was a hypothetical situation and new models would not necessarily be implemented. The project was a partnership with a health authority and was approved as a service improvement initiative by NHS Highland Ethics Committee.

Community is defined here as the people living within a more or less bounded territory (islands/peninsulas) – that is community as people in place. This aligns with Cohen’s [36] notion that community members have something in common with each other, but also distinguish themselves from members of other communities. In this study, some participating community members were also service providers, including healthcare workers. In participation terms, this was designed as citizen, rather than consumer, participation [24].

### Community selection

Four communities defined as remote [11] (<3,000 population and >60 minutes’ drive from a settlement of 10,000) with healthcare delivery models defined as ‘fragile’ by the health authority, were purposively selected (by health authority managers) for the study (see Table 1); two (A and B) were island and two (C and D) peninsula communities. Fragile communities were those where services depended on one or two key local health practitioners who were likely to leave in the next two years, due to retirement or job moves.

**Table 1:** Community characteristics

	**Community A**	**Community B**	**Community C**	**Community D**
Population Size in 2008/2009	206	126	483	150
% aged > =65	9.7	25.4	22.7	17.1
Approximate distance (time) from nearest District General Hospital by most common travel means	3 hours by ferry	2.5 hours by ferry	2.5 hours by car	2.5 hours by car
Distance to nearest GP practice	In situ	In situ	50 mins drive	50 mins drive
Top 5 issues for which local people attended local general practice (based on 2008/2009 QOF data)	Smoking related conditions	Smoking related conditions	Smoking related conditions	Smoking related conditions
	Hypertension	Hypertension	Hypertension	Hypertension
	Obesity	Obesity	Obesity	Obesity
	Depression	Depression	Depression	Depression
	Hypothyroidism	Asthma	Asthma	Hypothyroidism

Data obtained from local GP practice, local authority website, or QOF (Quality & Outcomes Framework) website.

Two communities had high proportions of over 65 s (relative to the Scottish average of 17% [37]). All are distant from major emergency and specialist health services. Distance is exacerbated by poor roads, sporadic mobile phone and broadband coverage, often adverse weather and sea-crossings - causing accessibility challenges. For the island communities, their remoteness has been countered by having resident single-handed GPs, but this model is regarded as unsustainable [19]. The four communities share consistent common conditions for consulting general practice.

### Data collection

Following introductory meetings in each community, a series of four workshops was held (see Figure 1) in the village hall. Workshop foci were: 1) *Future health*: identifying the role of health in the community’s future and comparing this with current health assets and challenges; 2) *Health evidence*: Comparing data about community health with local perceptions; 3) *Service roadshow:* presentations from experts, including health service and voluntary organisations’ employees, about service innovations and initiatives; 4) *Planning exercise*: where community members identified health priorities, competencies and infrastructure required to address them, and then designed a local service model to fit within the existing budget. The design and use of a game format in the planning exercise has been discussed previously [38]. Throughout the workshops, the central ideas of health (described as about physical, mental and social wellbeing [39]) and the competencies required to address health priorities were emphasised rather than structures and institutions (doctors and nurses, health centres and hospitals). This was because we wanted to start the conversation about health issues and then move to solutions in an open, objective way - rather than commencing with discussion about traditional service ‘solutions’.

Community members became involved by: a) being nominated by a local organisation or service, as researchers asked these to identify a citizen to participate in a group to discuss community health issues; or b) self-volunteered attendance, as the process and workshops were advertised widely and participation invited, using community noticeboards and websites, newsletters and newspapers.

Workshops were intended to be interactive, with two researchers (usually AN & JF) facilitating. The local NHS manager was generally present at each workshop. As well as participating in discussion, they were a useful reference source for technical questions e.g. how many hours is a community nurse ‘allowed’ to work per week? What is the scope of practice of a paramedic?

This paper draws on thematic and summary notes from the RSF workshops. Summary notes, rather than verbatim recordings, were taken as it was important to keep the discussion as natural as possible to facilitate participation. Notes were written on flip-charts as workshops progressed, with themes and outcomes summarised at the end. Conscious that there were those who would not, or could not, attend workshops, the researchers also conducted interviews between workshop stages with community members who either contacted directly or were suggested to researchers by service practitioners. A total of 39 informal interviews were conducted with people who did not wish to attend workshops, were disabled or housebound. Interviews tended to focus on the topics of the first two workshops – i.e. the role of health and discussion of local health priorities. Formal evaluation feedback on each workshop was elicited using short questionnaires. Numbers attending varied over the four workshops for each community: A) 3–30; B) 5–30; C) 8–28; D) 6–15, workshop attendees. There was no consistent pattern as to which workshop was best attended, with some sites having higher participation at the start and others having highest participation at Workshop 3. The workshop process occurred over 12 months for each community and all workshops took place within a 17 month period.

### Data analysis

Themes and decision-making points from workshops were summarised and fed back to participants for verification. Where ranking or selection exercises were conducted, these were recorded. Each workshop’s findings were summarised and reported to the wider community using newsletters, websites and community councils, giving further opportunities for comments and verification. Some interview data were hand-written and others audio-recorded depending on the choice and consent of interviewees and degrees of confidentiality (sometimes other family members, carers or friends were present, meaning recording was inappropriate.) Interview data were incorporated into findings.

## Results

### Findings

This paper focuses on workshop discussions so it describes themes arising, decision points, rankings and selections. This section follows the structure of the RSF workshop process.

### Future health

Community members were encouraged to talk about the role of health in the future of their communities and community health assets and challenges (summarised in Table 2). A strong future was described as requiring young families living locally and this meant that local employment opportunities were important. Health and healthcare had a role as young families and employers would be attracted to places that were perceived to be healthy, vibrant and had local health services. A community where older people could live with quality of life until they died, was another aspiration.

**Table 2:** Community health assets and challenges

**Common assets**	**Common challenges**
Community spirit, people look out for each other	Fears for security in an emergency situation due to remoteness/weather
Resourceful, adaptable community members	Older people have to leave community if their care needs become too great
Low crime, beautiful scenery, a safe place to raise children	Lack of affordable housing
More online working has allowed working people to settle in the communities	Current practitioner about to retire, concern about finding replacement
Personalised continuous care from local practitioners	Current practitioner provides “above and beyond the call of duty”: fear that replacement will not provide a similar service if not contractually obliged
Local health practitioners are social assets and provide preventative care	For practitioners providing 24/7 service, concern of insufficient support, issues of stress and isolation
Flexible, resourceful health practitioners who think and act ‘out of the box’ when necessary	Poor access to patient transport to outpatient facilities in distant hospitals
Responsive air ambulance service connecting community to acute care in emergencies	Confusion about current health services provision: who does what, who to call, when

### Health evidence

Asked to assess biggest local health problems, as they perceived them, community members consistently cited emergency and after-hours call-outs, cancer and alcohol abuse. When presented with anonymised data on the most common conditions for which community members visited their local GP practice (see Table 1), workshop participants were surprised at the prevalence of conditions that they perceived local people could prevent and address, including smoking and obesity. At workshop 2 conclusion, participants were asked to identify local healthcare priorities.

Consistency was found across communities, with priorities: what to do in emergency situations and how to recognise different levels of emergencies; how to improve local health and well-being, particularly preventing and managing chronic conditions; ensuring that older and vulnerable people could live in the community and that crises were anticipated; developing volunteering schemes that could help with transport, basic social support, first response and health promotion. This led to identifying services and initiatives that citizens would like to learn more about.

### Service road show

Workshop 3 involved inviting service providers and representatives of different initiatives to speak with communities. This gave community members opportunities to learn about innovations and ask questions. Community members either identified specific services they wanted to hear from or suggested health issues which researchers then investigated to identify initiatives that community members might want to hear about. Researchers found out about initiatives through internet searching (looking particularly for example initiatives in remote or rural communities), local service managers or national government departments and agencies. Thus, these were presented at service roadshows at one or more communities: NHS 24; tele-health and tele-monitoring; volunteering schemes, including lay first responders, time-banks, community transport; health worker roles, including physician assistants, generic health assistants, community nurses, nurse practitioners and paramedics. Community members said it was valuable to meet service providers and representatives of initiatives and to ask questions. They thought this allayed fears, provided ideas and helped to understand the activities of different services.

### Planning exercise

The final workshop was designed as a game, with cards, instructions and worksheets [38]. It involved stages of designing a service model:

1. Agree local health priorities.

2. Using provided lists of competencies (compiled from existing healthcare and volunteering role descriptions and some additional competencies that researchers constructed in response to previous workshop discussion), identify the top 10 competencies required to address local health priorities.

3. Align competencies with existing or new roles to design a local workforce. This had to comply with: a) a set of rules based on legislation, regulation and registration issues; e.g. what was within a particular health practitioners’ scope of practice or legal working hours; and b) a set budget (an approximation of the community’s current health and social care budget).

4. Identify other things needed to address priorities.

The top 10 competencies recurred for communities B, C and D. These were: basic technical skills, including taking temperature and pulse; minor injury and illness treatment; basic emergency aid; works after-hours; provides health/medical care home visits, when necessary; undertakes specialised care tasks in the home; terminal illness care; dispenses medicines; has intimate knowledge of the community so they can tell if something is amiss and act; supports mothers and young babies.

Only three participants attended Community A’s final workshop. We informally asked several previous participants why they had not attended. One reason given was the poor weather on the day and the old, damp village hall venue. However, others suggested that participation had been discouraged by one or more community members, who portrayed attendance as compliance with assisting the health authority to change local services. Researchers had also previously received an email from a community member stating that, on behalf of the community, he/she was stating that the community did not want to participate.

During the process and culminating at the final workshop, community participants expressed what we have come to regard as a set of key shared principles about their desired local healthcare model. These are summarised based on themes raised at workshop 4:

1. The healthcare worker(s) should reside and work locally. This provided health security, understanding of local context, continuity and made people feel that, although living in a remote place they were valued by statutory authorities. Living locally was thought to harness commitment and responsibility from a practitioner.

2. Expert emergency triage must be available locally. There was concern that lay first responders would be inexpert in discerning levels of emergency. There was demand for community level knowledge (e.g. through a printed algorithm supplied to remote community members) about levels of emergency situation, how to identify them and actions in response to each.

3. Anticipatory care and monitoring must be available locally. This related to high proportions of older people, often living isolated and/or alone and a desire to keep them living in their ‘home communities’. While good neighbours have a general support role, a formal role in anticipating care needs before a crisis, was identified. This was not necessarily seen as a highly skilled position, but could be partly fulfilled by volunteers or health assistants.

4. Leadership for local community health improvement and mustering volunteering as part of this, was desirable. Key aspects were generating ongoing volunteering; e.g. for first response; and knowledge about useful activities that citizens could implement, to improve local health and wellbeing.

Table 3 compares new designs with original models. Although participants from Community B discussed a range of new roles, they ultimately selected to replicate the existing model. Community C included new nurse practitioner and healthcare assistant roles and also included volunteering, while Community D invented a new combined nursing/paramedic role and were keen to involve non-health workers and to establish volunteer first responders.

**Table 3:** New and old service models

	**Community A**	**Community B**	**Community C**	**Community D**
Service model at start of study	1 ft GP	1 ft GP	Access to GP practice in neighbouring in larger village 50 mins drive away. Weekly local surgeries (half day), peripatetic nursing service available	Access to GP practice in neighbouring small community, 50 mins drive away. Nursing team with 2 locally based ft nurses, various carers
	2 pt nurses; one of these also does social care	1 pt nurse Various pt carers		
Model designed	Insufficient participants attended final workshop	1 ft GP pt district nurse, 3 pt care workers (including some intensive care hours) with some budget left for contingencies	1 ft Nurse Practitioner (working 24/7)	New resident practitioners with these skills & roles:
			Health care assistant	• Health/emergency care worker.
			5 hours per week of an Intensive Home Carer	• Non healthcare worker(s) to lead community health activities.
			A volunteer scheme for community carers, A first responder scheme Some budget left for contingencies	Volunteer first responder scheme to provide basic aid and emergency life support

ft = full-time, pt = part-time.

## Discussion

Findings show that using community participation can lead to designing new service models that fit within existing budgets and address local aspirations and healthcare priorities.

Participant communities were similar on dimensions of rurality, health status, aspirations and health priorities. They proposed a consistent set of requirements for remote Scottish primary healthcare. These were: resident practitioner(s), expert emergency triage, monitoring and anticipatory care of vulnerable people, community volunteering for health improvement, and leadership of community (health) volunteering.

Despite community similarities, community participation led to different service designs for three communities and one community did not participate at the final service design stage. These diverse outcomes suggest the influence of local contextual factors. This implies that different communities do want different things so suggesting the same standard model for all, apparently alike, rural communities is likely to be unsatisfactory and could lead to community disaffection. Community participation appears to allow for the customisation of local service models.

The two innovator communities (C and D) included new types of practitioners and community members as volunteers in their service designs, showing that some communities will be quite bold in a community participation process. Community B did not innovate and Community A informally withdrew. While reasons for this behaviour were not explicitly investigated, the literature and previous research suggest a range of explanatory ideas. Previous study has noted that communities are at various stages of receptivity for participation and that some maybe prone to hegemonic power, with prominent local people exerting influence over others [40]. For Community A, there was evidence of community leaders spreading advice not to participate as this would indicate compliance with health service change.

While Community B participated, the outcome replicated their current GP-led model, despite much discussion at their workshops about the value of different types of workers. Actions of A and B could be interpreted as forms of protest or at least non-compliance with, the potential for change.

Communities A and B had ‘rich’ existing service delivery models, each having a resident GP and a nurse despite small populations. A and B are island communities and thus may feel strong insecurity, particularly they might perceive threats to health and community sustainability if they lost their GP. Conversely, C and D may have been more receptive to change due to circumstances. Residents of C were dissatisfied with having no locally resident health professionals and were keen to advocate for improvement in local provision. Community D was about to lose the resident nurse and people were anxious about local service depletion. These circumstances may have made Communities C and D readier to envision innovative services, particularly as their designs highlight local presence as a priority. Community receptivity for change can be understood to arise from different origins. Portes [41] reflects on relationships between local social capital and change, with strong bonding capital (i.e. relationship ties between neighbours that are alike in social group and status, and embedded in the local community) less associated with innovation, compared with the existence of strong local bridging capital (i.e. relationship ties between people in the community and people with access to external resources) which is more associated with innovation [41]. Although we lack evidence about length of time community participants had lived locally, observation in all of the communities indicates that they combined diverse long-term locals and incomers and this was reflected in workshop attendance. This questions the role of social capital as underpinning different responses. Rather, we suspect evidence from a previous study about how remote health services incrementally adapt over time, in relation to their context, might be relevant [42]. Findings of that study indicated that rural health services developed incrementally in relation to inter-linked demand and supply factors of what skills and roles were available locally and how local ‘consumers’ adapted to these. We suspect community members need to be able to envisage and therefore accept, change to aspects of service delivery that are akin to current or known services, as opposed to being asked to envision radical change. That is, Community C wanted moves back to a previous model of a resident community nurse because they remembered this and nurses still lived in the community (though now working peripatetically). Community D saw nurses and paramedics already in their community and thus could envisage a role that combined these two sets of skills. Although we suggest this as a theory, why communities design different models is an issue worthy of future, in-depth exploration.

The composition of the participant group is likely to impact both on choices made, and acceptance of these by the wider community. For all communities, only small proportions of the population attended workshops and outcomes depended on their views. Thus, if the models designed were to become more than hypothetical – and moved to implementation, fellow citizens might protest that they had not participated in decision-making. This issue was raised by a health manager who questioned the status of the designed models and community reactions if the health service moved to implementation? “When and how is a community decision made?”, she asked. Inclusion is discussed in community participation literature, with questioning about the credibility of processes involving small numbers. Others suggest that all are offered the opportunity to participate and that not participating is also a choice [43]. Taylor et al. [21] note that community participation implies collective involvement and Alford [24] that true citizen involvement requires representative participants. In practical terms, methods such as Renn and colleague’s citizen panels [44], seem to come closest to an ideal inclusion method. They applied random selection to lists of community residents to invite citizens to participate in community decision-making forums. They say this is a way to ensure marginalised people are invited to participate, but there is still the problem of whether such people feel sufficiently comfortable to turn up in a public setting.

In redesigning rural services, standard models devised for communities with shared characteristics avoid these complications of involving communities that we have described. Their basis is to produce equity for all – “…not ad hoc responses…” [3]. If community participation results in different service designs for apparently similar communities, this could result in some communities being overworked compared with others. An example would be if local people provided lay first response in some communities, but not in others. Hanlon and Halseth [45] have commented on the exhaustion in rural Canadian communities due to service withdrawal and community members having to co-produce services.

But while the acceptability of diversity, with potentially different impacts for local residents, presents a philosophical debate, in fact there are uneven models already present in Scottish remote healthcare, and likely internationally. Taking the example of first response, some communities have already established volunteer first responder schemes, while others have not [46]. Community service models vary as do the skills and experience of individual health professionals [47]. Some recent initiatives in rural health endorse the perspective that it is realistic to draw on existing assets, local needs and rural propensity for adaptation to circumstances, when redesigning local services [48]. As co-production, resilience and resourcefulness become increasingly promoted in government policy, it seems communities will have to increasingly become self-reliant co-producers, with the implications for diverse service accessibility and potentially for outcomes, that brings [49]. The challenges of community participation may make the application of standard models seem attractive when redesigning services; however, there is evidence that rural communities protest if externally conceived plans, into which they have had no input, are inflicted upon them [50,51].

The overall RSF study was about producing a community participation process for remote places that could be used by health authority managers (i.e. was straightforward to understand, produced plans for service redesign, and was relatively cheap). It thus collected limited, mainly observational and informal, data about participants, their motivations and experiences. As engaging people in a comfortable and ‘normal’ process was important, most data were not recorded verbatim, meaning that the words of participants cannot be shared here. Since the process was collaborative, it was necessary to ‘go with the community’ , rather than being overly directive.

This produced messy evidence, but Tritter and McCallum [52] note that “users must have agency and the ability to shape the methods used for their involvement”. We do not have formal data about why people participated or not. This would have been useful regarding the apparent withdrawal of Community A at the design stage. As the models derived by communities were hypothetical, we do not know what would happen if the health authority chose to work with the communities to implement the designs. However, in enacting an actual process of community participation to produce designs for future services, a number of issues about rural community participation have been raised.

## Conclusions

This paper illustrates that community participation can be used to design rural primary healthcare services, but outcomes may vary from innovative models to passive protest, depending on community receptiveness. That communities produced different responses to apparently similar circumstances and priorities, suggests that aspects of local context affect the choices communities make and therefore that engaging community members should add value when designing acceptable local services. Population health planning incorporates community participation while top-down standard models appear to neglect a community perspective, even though they are underpinned by a desire for fairness. There maybe a role for standard models as part of community participation discussions as they are informed by evidence and provide abstractions that community members can use as a basis for discussing adaptations or additions that they deem important given local requirements.

Inviting communities to participate in decision-making produces messy and unpredictable outcomes and this is insufficiently acknowledged by policymakers. More needs to be written on the productive and/or messy aspects of community participation in system change.

## Competing interests

The authors declare that they have no competing interests.

## Authors’ contributions

JF conceived of and led the RSF study, participated in study design, assisted with data collection and analysis and led on writing this paper. AN organized workshops, led on data collection and analysis and assisted in writing this paper. Both authors read and approved the final manuscript.

## Pre-publication history

The pre-publication history for this paper can be accessed here:

http://www.biomedcentral.com/1472-6963/14/130/prepub
